# Sodium Tanshinone IIA Sulfonate as a Potent IDO1/TDO2 Dual Inhibitor Enhances Anti-PD1 Therapy for Colorectal Cancer in Mice

**DOI:** 10.3389/fphar.2022.870848

**Published:** 2022-04-27

**Authors:** Rongjie Zhang, Yuanfeiyi Wang, Dan Liu, Qing Luo, Peixin Du, Haiyan Zhang, Wenshuang Wu

**Affiliations:** ^1^ Laboratory of Integrative Medicine, Clinical Research Center for Breast, State Key Laboratory of Biotherapy, West China Hospital, Sichuan University and Collaborative Innovation Center, Chengdu, China; ^2^ School of Pharmacy, Sichuan Industrial Institute of Antibiotics, Chengdu University, Chengdu, China; ^3^ Sichuan Provincial Maternity and Child Health Care Hospital, Chengdu, China; ^4^ The Affiliated Women’s and Children’s Hospital of Chengdu Medical College, Chengdu, China; ^5^ Department of Thyroid Surgery, West China Hospital, Sichuan University, Chengdu, China; ^6^ Laboratory of Thyroid and Parathyroid Disease, Frontiers Science Center for Disease-Related Molecular Network, West China Hospital, Sichuan University, Chengdu, China

**Keywords:** sodium tanshinone IIA sulfonate, IDO1, TDO2, treg, immunotherapy, colorectal cancer

## Abstract

Although the antitumor efficacy of immune checkpoint blockade (ICB) has been proved in colorectal cancer (CRC), the results are unsatisfactory, presumably owing to the presence of tryptophan metabolism enzymes indoleamine 2,3-dioxygenase 1 (IDO1) and tryptophan 2,3-dioxygenase 2 (TDO2). However, only a few dual inhibitors for IDO1 and TDO2 have been reported. Here, we discovered that sodium tanshinone IIA sulfonate (STS), a sulfonate derived from tanshinone IIA (TSN), reduced the enzymatic activities of IDO1 and TDO2 with a half inhibitory concentration (IC_50_) of less than 10 μM using enzymatic assays for natural product screening. In IDO1- or TDO2- overexpressing cell lines, STS decreased kynurenine (kyn) synthesis. STS also reduced the percentage of forkhead box P3 (FOXP3) T cells in lymphocytes from the mouse spleen cocultured with CT26. *In vivo*, STS suppressed tumor growth and enhanced the antitumor effect of the programmed cell death 1 (PD1) antibody. Compared with anti-PD1 (α-PD1) monotherapy, combined with STS had lower level of plasma kynurenine. Immunofluorescence assay suggested that STS decreased the number of FOXP3+ T cells and increased the number of CD8+ T cells in tumors. Flow cytometry analysis of immune cells in tumor tissues demonstrated an increase in the percentage of tumor-infiltrating CD8+ T cells. According to our findings, STS acts as an immunotherapy agent in CRC by inhibiting both IDO1 and TDO2.

## Introduction

Colorectal cancer (CRC) is one of the most common tumors worldwide and leads to approximately 8.6% of cancer-related deaths in China (Feng et al., 2019; Yu et al., 2022; Zheng et al., 2022). The incidence of CRC has a strong association with age. It has increased in those under 65 years old and tends to be younger (Benson et al., 2021). Regarding CRC treatment, unlike melanoma, renal cancer, bladder cancer, and lung cancer, only a subset of patients respond to immunotherapy depending on the level of tumor mutation burden (TMB). In a phase III, randomized, open-label KEYNOTE-177 study of 307 patients with mismatch repair-deficient or microsatellite instability high (dMMR/MSI-H) CRC, the median progression-free survival (PFS) in the pembrolizumab group was more extended than in the group who received first-line chemotherapy (Andre et al., 2020). Nevertheless, immunotherapy is ineffective for patients with mismatch repair-proficient or microsatellite stable (pMMR/MSS) CRC. In addition to TMB, the composition of the tumor immune microenvironment (TIME) is another determinant of the response to immunotherapy, such as immunosuppressive cells (regulatory T cells and myeloid-derived suppressor cells) and cytokines (PGE2, IDO1, TGF-*β*, IL-10, etc.) (Liu and Cao, 2016).

Indoleamine 2,3-dioxygenase 1 (IDO1) is overexpressed in numerous tumors, including melanoma, breast cancer, and CRC (Chen et al., 2007; Soliman et al., 2013; Qian et al., 2016). It mediates tumor development and progression by catalyzing the cleavage of tryptophan and promoting the production of immune-suppressive factors, such as kynurenine (Amobi et al., 2017). Tryptophan deficiency induces cell cycle arrest and apoptosis in T cells (Lee et al., 2002). Meanwhile, the accumulation of kynurenine promotes aryl hydrocarbon receptor (AHR) nuclear translocation, which can accelerate tumor escape from immune surveillance by triggering the generation of immunosuppressive cells (Zhang et al., 2021). As a consequence, pharmaceutical suppression of IDO1 has significant antitumor potential. To date, numerous small molecule-selective IDO1 inhibitors have been studied in clinical trials to treat advanced cancers, such as epacadostat, BMS-986205, PF-06840003, indoximaod, NLG802, and LY3381916. Apart from those, various peptide vaccines targeting IDO1 have been tested in clinical research to assess their efficacy in cancer therapy (Liu et al., 2018). It is worth noting that the proteolysis targeting chimera (PROTAC) technique has been used to degrade IDO1 as a strategy for cancer immunotherapy (Hu et al., 2020). In regard to these inhibitors, it has been proven that IDO1 inhibitors, such as 1-methyltryptophan (1-MT) and epigallocatechin gallate, can act synergistically with immune checkpoint inhibitors to enhance their antitumor efficacy in CRC (Shi et al., 2021). However, tumor cells and myeloid cells may express tryptophan 2,3-dioxygenase 2 (TDO2) and catabolize tryptophan *via* an alternative pathway that replaces or complements IDO1. Thus, developing dual inhibitors of IDO1 and TDO2 might be an effective method for cancer immunotherapy.

Natural products have been regarded as an essential source of novel drug discoveries for a long time. Several natural compounds with IDO1 or TDO2 inhibitory activity have been discovered during the last two decades. For example, Exiguamine A, isolated from the marine sponge Neopetrosia exigua, has a Ki of 210 nM for inhibition of IDO1 *in vitro* (Brastianos et al., 2006). Moreover, the inhibitory activities of tanshinone derivatives and naphthoquinone on IDO1 have also been reported (Pan et al., 2018; Guo et al., 2020). Therefore, it is preferable to discover dual inhibitors of IDO1/TDO2 from natural products or derivatives.

Recently, combination therapy with multiple regimens has become a promising strategy for cancer treatment, such as MAPKi and phototherapy therapy combinations with PD1 antibody treatment for melanoma (Liu et al., 2021). In CRC, small molecules targeting the MAPK pathway in combination with immune checkpoint blockers, photothermal therapy in combination with immune checkpoint blockers (Wang et al., 2021), and so on, have been studied. All of them have demonstrated synergistically improved therapeutic efficacy. Our study described the inhibitory effect of tanshinone IIA sulfonate (STS), a sulfonate derived from tanshinone IIA (TSN), on IDO1/TDO2 and tested the antitumor activity of STS combined with anti-PD1 to treat CRC *in vivo*, indicating that STS could prevent the progression of CRC and improve the efficacy of anti-PD1 therapy.

## Materials and Methods

### Cell Culture

Human embryonic kidney 293T cells were cultured in DMEM. The mouse colorectal carcinoma cell line CT-26 and the acute leukemia cell line Jurkat were cultured in RPMI-1640 medium. Both media were supplemented with 10% fetal bovine serum and 1% penicillin/streptomycin in a humidified 5% CO_2_ incubator at 37°C.

### Recombinant Protein Expression and Purification

A 6X His tag for purification and a peptide of adenylate kinase (AK sequence: RIILLGAPGAGKGTQAQFIMEKYGIPQISTGDMLRAAVKSGSELGKQAKDIMDAGKLVTDELVIALVKERIAQEDCRNGFLLDGFPRTIPQADAMKEAGINVDYVLEFDVPDELIVDRIVGRRVHAPSGRVYHVKFNPPKVEGKDDVTGEELTTRKDDQEETVRKRLVEYHQMTAPLIGYYSKEAEAGNTKYAKVDGTKPVAEVRADLEKILG) to improve the yield were added at the N-terminus of proteins IDO1 and TDO2 (His-AK-IDO1 or His-AK-TDO2) (Luo et al., 2016). The primers (F1: cgc​ata​tgg​gca​gca​gcc​atc​atc​atc; R1: cca​tag​ctg​gag​ctt​tat​cat​cat​cat​cac​cag​aac​cac) were used for PCR of His-AK from the vector provided kindly by Dan Luo. The primers (F2: tag​tgg​ttc​tgg​tga​tga​tga​tga​taa​agc​tca​cgc​tat​gga​aaa​ctc​ttg; R2: cgc​agc​tct​taa​cct​tct​ttc​agc​aga​gat​ttt​tc) were used for clone IDO1. For clone His-AK-TDO2, the primers (F1: cgc​ata​tgg​gca​gca​gcc​atc​atc​atc; R1: ttt​atc​atc​atc​atc​acc​aga​acc​act​a) were used for PCR for His-AK, and the primers (F2: tag​tgg​ttc​tgg​tga​tga​tga​tga​taa​aag​cgg​gtg​tcc​gtt​ttt​agg​g; R2: cgg​agc​tct​taa​tcg​ctt​tca​tcg​ctg​cta​aaa​tag) were used for clone TDO2. Two fusion fragments were cloned into PET25b digested with NdeI and Sacl. After 0.5 mM isopropyl-β-D-thiogalactopyranoside (IPTG) induction at 16°C for 8 h, the cells were harvested by centrifugation at 8,000 rpm/min for 5 min. To increase the expression of TDO2, 40 μM hemin was added to the Luria-Bertani (LB) medium when induced with IPTG. After centrifugation, the cell pellet was resuspended in an appropriate volume of lysis buffer [50 mM Tris-HCl, pH = 8.0, 100 mM NaCl, 5 mM EDTANa_2_, 2 mM DTT, cell pellet mass (g): buffer volume (mL) was 1: 20] and lysed by sonication. Then, 13,500 rpm/min for 15 min was implemented at 4°C to remove cell debris, and the protein was solubilized in the supernatant. Protein purification was carried out using the Ni column (Bestchrom, Shanghai, China).

### Enzymatic Assays

The enzymatic assays were carried following the methods described in the reported procedures (Malachowski et al., 2016). Briefly, the 100 μL reaction mixture contained 0.05 M potassium phosphate buffer (PBS), pH = 6.5, 40 mM vitamin C, 0.2 mg/mL H_2_O_2_, 0.02 mM methylene blue, and 0.08 mM tryptophan, and with 100 μg/mL IDO1 or 200 μg/mL TDO2. First, the reaction solution and compounds were incubated for 30 min at 37°C. Then, the reaction was stopped with 80 μL of 30% (w/v) trichloroacetic acid at 65°C for 15 min. The solution was then centrifuged at 12,000 rpm/min for 10 min, and the supernatant was mixed with an equal volume of 2% (w/v) p-dimethylaminobenzaldehyde in acetic acid. Finally, the kynurenine in the yellow product was measured using a multifunction microplate reader (Synergy H1, BioTek, United States) at 490 nm.

### Cell-Based Assay for Inhibitor

To overexpress IDO1 and TDO2 in cells, the primers (F1: atg​gca​cac​gct​atg​gaa​a; R1: tta​acc​ttc​ctt​caa​aag​gga; F2: cac​cga​ctc​tag​aac​tag​tga​tgg​cac​acg​cta​tgg​aaa; R2: gcc​agt​aac​gcg​atc​gaa​ttt​taa​cct​tcc​ttc​aaa​agg​ga) were used to clone IDO1 into the CPPT-puro vector by digestion with BamHI and EcoRI. The primers (F1: atgagtg ggtgcccattttta; R1: tta​atc​tga​ttc​atc​act​gct​ga; F2: cac​cga​ctc​tag​aac​tag​tga​tga​gtg ggtgcccatt ttta; R2: gcc​agt​aac​gcg​atc​gaa​ttt​taa​tct​gat​tca​tca​ctg​ctg​a) were used to clone TDO2. The cell-based assay procedure were performed under the protocol described in the literature (Malachowski et al., 2016). Briefly, IDO1-or TDO2-overexpressing 293T cells were harvested and plated at a density of 2.5 × 10^4^ cells/well in a 96-well culture plate. Simultaneously, a serial dilution of STS in 50 μL culture media containing 100 μM tryptophan was added to the wells. After 24 h, 75 μL of supernatant was transferred to a new 96-well plate and mixed with 35 μL of 50% trichloroacetic acid in each well, and the plate was incubated at 65°C for 15 min turning formylkynurenine to kynurenine. The reaction mixture was centrifuged for 10 min at 13,000 g to remove the deposits. Then, 50 μL supernatant from each well was transferred to another 96-well plate and mixed with an equal volume of 2% p-dimethylaminobenzaldehyde in acetic acid. A microplate reader was used to measure the yellow reactions at 490 nm. Additionally, the cell activity was detected by the MTT assay following the transfer of supernatant.

### MTT Assay

To investigate the effect of STS on cell proliferation, 20 μL of 2.5 g/L MTT solution was added to each well of a 96-well plate and incubated at 37°C for 4 h. After lysing formazan in 100 µL of 10% (w/v) SDS solution, the absorbance at 570 nm was measured using a multidetection microplate reader.

### Western Blot

IDO1- or TDO2- overexpressing and normal 293T cells were washed with precooled PBS and lysed in RIPA solution with a protease inhibitor cocktail for 30 min on ice. After centrifuging at 13,500 g at 4°C for 15 min, the supernatant was quantified by BCA (Biyuntian, China) and denatured at 100°C for 10 min. SDS-PAGE was used to separate the protein samples. Then, the samples were transferred to a 0.22 μm PVDF membrane at 100 V for 90 min. After blocking the membrane with TBST containing 5% BSA for 1 h at room temperature, it was incubated with the primary antibodies overnight at 4°C and then with secondary antibodies for 1 h. Proteins were visualized using ECL reagent (Millipore, United States). Finally, the gel imaging system (Bio-Rad, United States) was used for imaging. The antibodies used for WB are listed below: rabbit monoclonal antibodies to TDO2 (Abcam, Cat # 259359, 1:1000 dilution), rabbit monoclonal antibodies to IDO1 (Abcam, Cat # 211017, 1:1000 dilution), and mouse monoclonal antibodies to GAPDH (Zhongshan Jinqiao, 1:1000 dilution) as the reference.

### Molecular Docking

ChemBio3D Ultra 14.0 (Cambridge Soft, Cambridge, MA, United States) was adopted to construct the structures of IDO1 and TDO2. Then, energy majorization of the molecular structures was performed using the MM2 force field such that the convergence condition of the root mean square (RMS) was less than 0.0001 kcal mol^−1^ Å^−1^. Following an initial minimization of the molecule, the restrained electrostatic potential (RESP) charges of the molecule and precise optimization [at the b3lyp/6-311++G (d,p) level] calculations were performed using the Gaussian 09 software combined with the multiwfn software (Frisch et al., 2009; Lu and Chen, 2012). After optimization, the optimized molecules were docked to the binding pockets of the receptor by the AutoDock4.2 software package based on the Lamarckian genetic algorithm (LGA), which evaluated docking results by searching for conformations and the semiempirical free energy scoring function (Hou et al., 2013). In this work, we built a 50 Å × 50 Å × 50 Å rectangular box with a grid space of 0.375 Å, and the box centers of IDO1 (PDB entry: 2D0T) and TDO2 (PDB entry: 2NW8) were set as x: 58.506/40.593, y: 52.978/−48.557 and z: 16.523/30.523, respectively. The GA-LS running set as 128 in all docking and the structure with the lowest energy in the largest cluster was taken as the final result.

### Animal Experiments

Six-week-old female BALB/c mice were purchased from Weitong Lihua Experiment Animal Technology (Beijing, China). All animal experiments were performed according to the protocols approved by the Ethics Review Committee of Animal Experimentation of Sichuan University. CRC cells CT26 (5 × 10^5^) in 100 μL D’-HANKS were inoculated subcutaneously into one flank of each mouse to examine the therapeutic potential of STS and STS combined anti-mouse PD1 [Gifted by Conmed Biosciences Inc. (Chengdu, China)] on antitumor immune responses *in vivo*. When the tumor size reached ≈80 mm^3^, mice were randomized into four treatment groups (*n* = 5 per group). The mice received intraperitoneal injections of the following drugs: 1) saline; 2) STS (20 mg/kg, q.d.); 3) anti-PD1 (3 mg/kg, q.w.); and 4) STS (20 mg/kg, q.d.) + anti-PD1 (3 mg/kg, q.w.). Tumor volume was measured every 2 days using a Vernier caliper and computed using the formula: volume = (tumor length) × (tumor width) ^2^/2. On day 27, tumors were collected for flow cytometry and IF analysis. ELISA was used to measure kynurenine in plasma and IFN*-γ* in tumors.

### Immunofluorescence Staining

The tumor fragments were fixed in 10% formalin before being embedded in paraffin. Paraffin slides were then deparaffinized with xylene and ethanol. Tumor tissues were cut into 3 μm pieces and incubated at 62°C for 4 h. Heat-mediated antigen retrieval was performed in EDTA buffer (pH = 9.0) for 16 min, followed by 15 min in 3% hydrogen peroxide, and then overnight in a wet container at 4°C with primary CD8 antibody (Abcam, Cat# 217344, diluted 1:2000). Furthermore, the paraffin sections were treated with CD4 antibody (Abcam, Cat# 183685, diluted 1:1000) and FOXP3 antibody (Invitrogen, Cat# 14577380, diluted 1:100) after being permeabilized with 0.3% Triton X-100 for 15 min. Relevant secondary antibodies conjugated with Alexa Fluor 488 (Invitrogen, Cat# A21206, diluted 1:400) and Alexa Fluor 594 (Invitrogen, Cat# A48264, diluted 1:400) were added and incubated for 1 h in darkness at room temperature. Hoechst 33342 (Biyuntian, Cat# C1011) was added to a final concentration of 10 μg/mL (Biyuntian, Cat# C1011) and incubated for 10 min at room temperature in the dark to stain the nucleus. Nikon Ni-E was used to capture immunofluorescence images.

### Flow Cytometry

To study the effect of STS on the proliferation of lymphocytes, the spleen was harvested from a normal BALB/c mouse after being sacrificed and processed with a 70 μm Nylon cell strainer (Biofil, Guangzhou, China). According to the manufacturer’s instructions, lymphocytes from the spleen were isolated with Mouse Lymphocyte Separation Medium (Dakewe Biotech Company Ltd., Shenzhen, China). Then, the lymphocytes were stained with 2.5 μM and 100 μL CFSE for 8 min at room temperature. Following staining, a 500 μL RPMI-1640 medium was added to block the CFSE-labeling reaction, and the cells were washed twice with a 500 μL RPMI-1640 medium. Next, 3 × 10^6^ lymphocytes per well were seeded into a 12-well plate that had been precoated with anti-CD3 antibody (Biolegend, Cat# 100359, 10 μg/mL). After cell seeding, the STS formulated with medium containing CD28 antibody (Biolegend, Cat# 102121) and IL-2 (Peprotech, Cat# 212-12) was added. After culturing for 48 h, the CFSE-positive cells were detected utilizing a flow cytometer.

To study the effect of STS on Tregs, the CT26 cells (1 × 10^5^/well) were seeded into a 12-well plate with 1 mL of medium containing 100 ng/mL of mouse IFN-*γ* (Peprotech. Cat# 315-05). In the meantime, the cells were treated with different concentrations of STS (0, 10, 25, 50 μM) and incubated at 37°C. After 24 h, the medium was withdrawn, and new lymphocytes were introduced with anti-CD3, anti-CD28, IL-2, and concentration gradient STS (0, 10, 25, 50 μM). After 48 h of coculture, the cells were harvested. Before analysis, cells were treated for 15 min at room temperature with the Zombie UV Fixable Viability Kit (BioLegend, Cat# 423108) to exclude dead cells. After washing, anti-CD45-APC-Cy7 (BioLegend, Cat# 103116) and anti-CD4-FITC (BD, Cat# 557307) were applied for a 30 min incubation at room temperature. The cells were then fixed and permeabilized using the True-NuclearTM Transcription Factor Buffer Set (BioLegend, Cat# 424401). Finally, the cells were incubated at room temperature for 30 min with anti-FOXP3-PE (Invitrogen, Cat# 12-5773-80). Following washing, the percentage of Zombie- CD45+ CD4+ FOXP3+ T cells was analyzed by flow cytometry.

To study the effect of STS on the percentage of cytotoxic T cells in tumors, tumor samples were cut into pieces with scissors and digested for 30 min at 37°C in RPMI 1640 containing 0.2 mg/mL collagenase type I (Gibco, Cat# 12-5773-80) and type IV (Gibco, Cat# 17104019). Tissues that had been digested were gently filtered through a 70 μm cell strainer. Single-cell suspensions were centrifuged for 5 min at 600 g/min and washed twice with D’-HANKS buffer. Then, anti-CD3-FITC (Biolegend, Cat# 100204, 1:100) and anti-CD8-PE (Biolegend, Cat# 553032, 1:100) antibodies were employed for cell staining at room temperature for 30 min. The cells were then stained with 7AAD Viability Staining Solution (Biolegend, Cat# 420404) for 5 min at room temperature before being analyzed by flow cytometry.

### Enzyme-Linked Immunosorbent Assay

To detect the concentration of kynurenine in plasma, we employed a mouse kynurenine ELISA kit (Yanjin Biology Company, Cat# F07945) for examination. First, after collecting approximately 600 μL of blood from mouse eyeballs, serum was collected by centrifugation at 500 g at 4°C for 5 min. Purer serum was obtained after an additional 15 min of centrifugation at 4000 rpm/min. Serum samples were determined after being diluted five times.

To measure the concentration of IFN-*γ* in tumor tissue, 0.1 g of the tissue block was weighed and placed in 1.5 mL EP tubes before adding PBS [tissue weight (g): PBS volume (mL) = 1:9]. Next, a small scissor was used to cut the tissues as soon as possible on ice. After grinding with a high-throughput tissue grinder (SCIENTZ-48, Zhejiang) at 70 Hz for 30 s at 10 s/time, the samples were centrifuging at 3000 rpm/min for 15 min. An ELISA kit (Biolegend, #1210002) was used to detect IFN-*γ* in the supernatant.

### Hematoxylin and Eosin staining

The hematoxylin and eosin (H&E) staining was performed according to standard procedures. Briefly, internal organ paraffin sections were dewaxed to water. Hematoxylin (Thermo, Cat# 7211) was used to counterstaining for 30 s, followed by rinsing with running water for 5–10 min to remove the blue color. After rinsing, 10 s with 1% hydrochloric acid alcohol before rinsing for 5 min with running water. Eosin (Thermo, Cat # 7111) staining was performed for 10 s, washed twice with running water for 5 min, dehydrated with graded alcohol, rendered transparent with xylene, and mounted with neutral gum. Nikon Ni-E was used to capture images.

### Statistical Analysis

One-way ANOVA analysis on GraphPad Prism 9.0.0 was used for statistical analysis in this work. The results were all reported as the mean ± SD. Statistical significance thresholds were set at *p < 0.05; **p < 0.01; ***p < 0.001; ****p < 0.0001.

## Results

### Sodium Tanshinone IIA Sulfonate Acted as a Dual Inhibitor of Indoleamine 2,3-Dioxygenase 1 and Tryptophan 2,3-Dioxygenase 2

**FIGURE 1:**
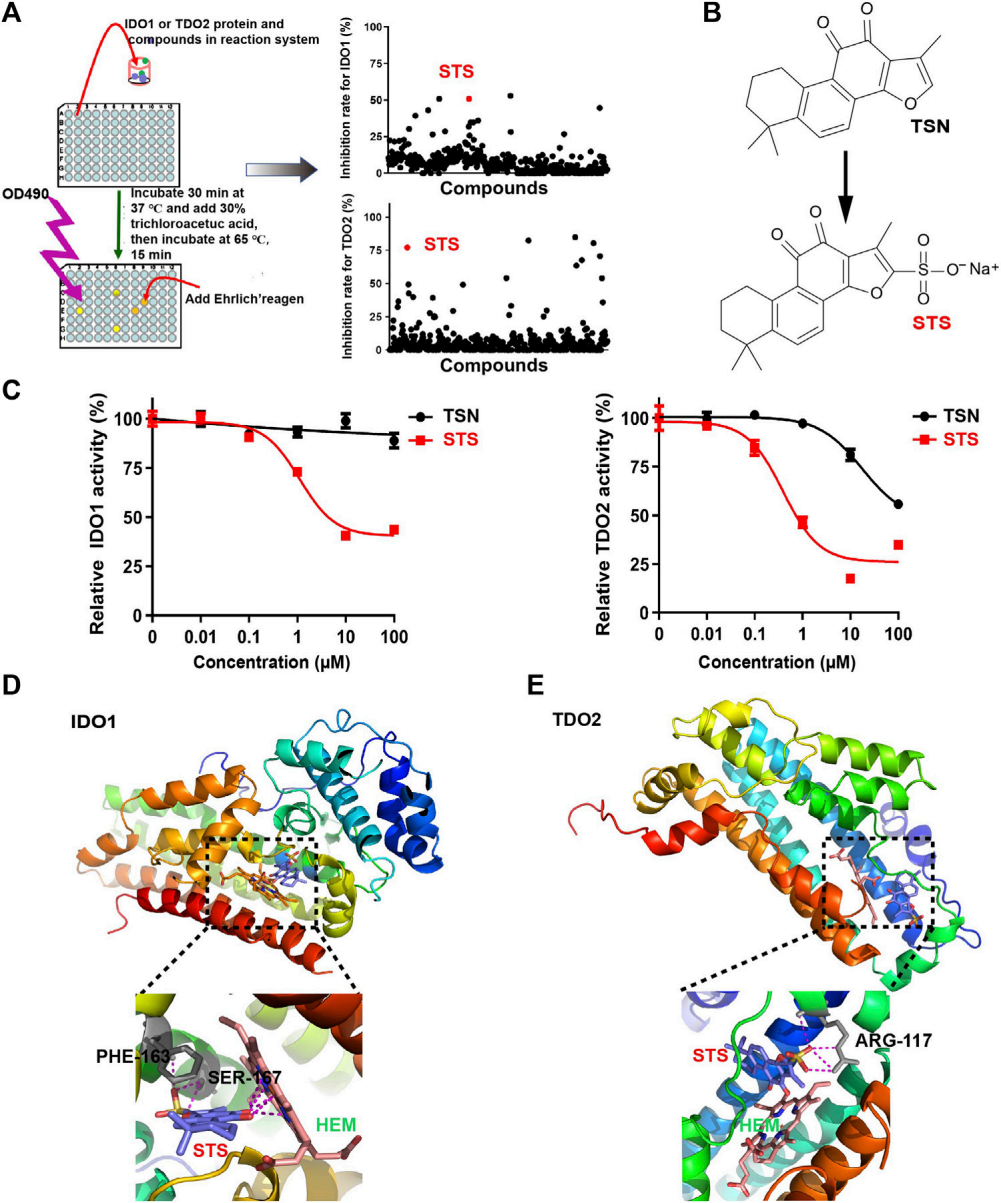
STS inhibited the enzymatic activity of IDO1 and TDO2 extracellularly. **(A)** The flowchart depicts the extracellular screening of IDO1 and TDO2 inhibitors. The concentration of all compounds was 10 μM. **(B)** The chemical structures of STS and TSN. The sulfonic acid group was used to improve the TSN’s hydrophilicity. **(C)** Evaluation of IDO1 and TDO2 enzymatic activity *in vitro*. STS or TSN was added at the indicated concentrations, with three replicates for each concentration. **(D,E)** Molecular docking of IDO1 or TDO2 with STS. Amino acid residues that interacted with STS are colored gray, and their names are colored black. STS is marked in red and hemin (HEM) is marked in green. Red in STS represents oxygen atoms. Yellow in STS represents sulfur atoms.

Considering the importance of IDO1 and TDO2 inhibitors in tumor immunotherapy, we developed an extracellular inhibitor screening model. First, we employed a prokaryotic protein expression system to produce the full-length IDO1 and TDO2 proteins. To facilitate purification, IDO1 and TDO2 carried an N-terminal His-tag. To improve the yield of IDO1 and TDO2, a peptide of *Escherichia coli* adenylate kinase (AK) was added between the His-tag and IDO1 or TDO2 proteins. Then, the proteins were purified using Ni-affinity chromatography. After that, the activities of IDO1 and TDO2 to catalyze the degradation of tryptophan were initially tested. Despite the presence of His-tag and AK peptide, the purified protein retained evident activity. Using the inhibitor screening approach described in the procedures (Figure 1A), we discovered that several compounds, including mangostin and androstenedione, could inhibit the activity of IDO1 (Supplementary Figures S1A,B), and tanshinone IA inhibited TDO2 activity (Supplementary Figure S1C). The IDO1 inhibitory activities of mangostin and androstenedione were firstly found by us. Remarkably, STS (Figure 1B), a derivative of TSN extracted from the dried roots of Danshen, could simultaneously inhibit the enzymatic activities of IDO1 and TDO2 (IC_50_ < 10 μM). Moreover, it was interesting to know that TSN only reduced the activity of TDO2 but not IDO1 (Figure 1C).

To investigate the molecular basis of the inhibitory activity, molecular docking analyses were conducted. STS was docked into the binding pocket of the crystal structure of IDO1 (PDB access code: 2D0T) (Sugimoto et al., 2006) or TDO2 (PDB access code: 2NW8) (Forouhar et al., 2007). As illustrated in Figure 1D, the carbonyl of STS interacted with the heme iron in the tryptophan-IDO1 binding pocket, which was critical for IDO1’s enzymatic activity (Basran et al., 2021). Furthermore, STS could interact with PHE-163 and SER-167, which may compete with tryptophan binding to IDO1 (Figure 1D). For TDO2, STS was only linked to ARG-117 (Figure 1E), which also influenced the coupling of tryptophan and TDO2 (Rafice et al., 2009).

### Sodium Tanshinone IIA Sulfonate Decreased the Generation of Kynurenine at the Cellular Level

**FIGURE 2:**
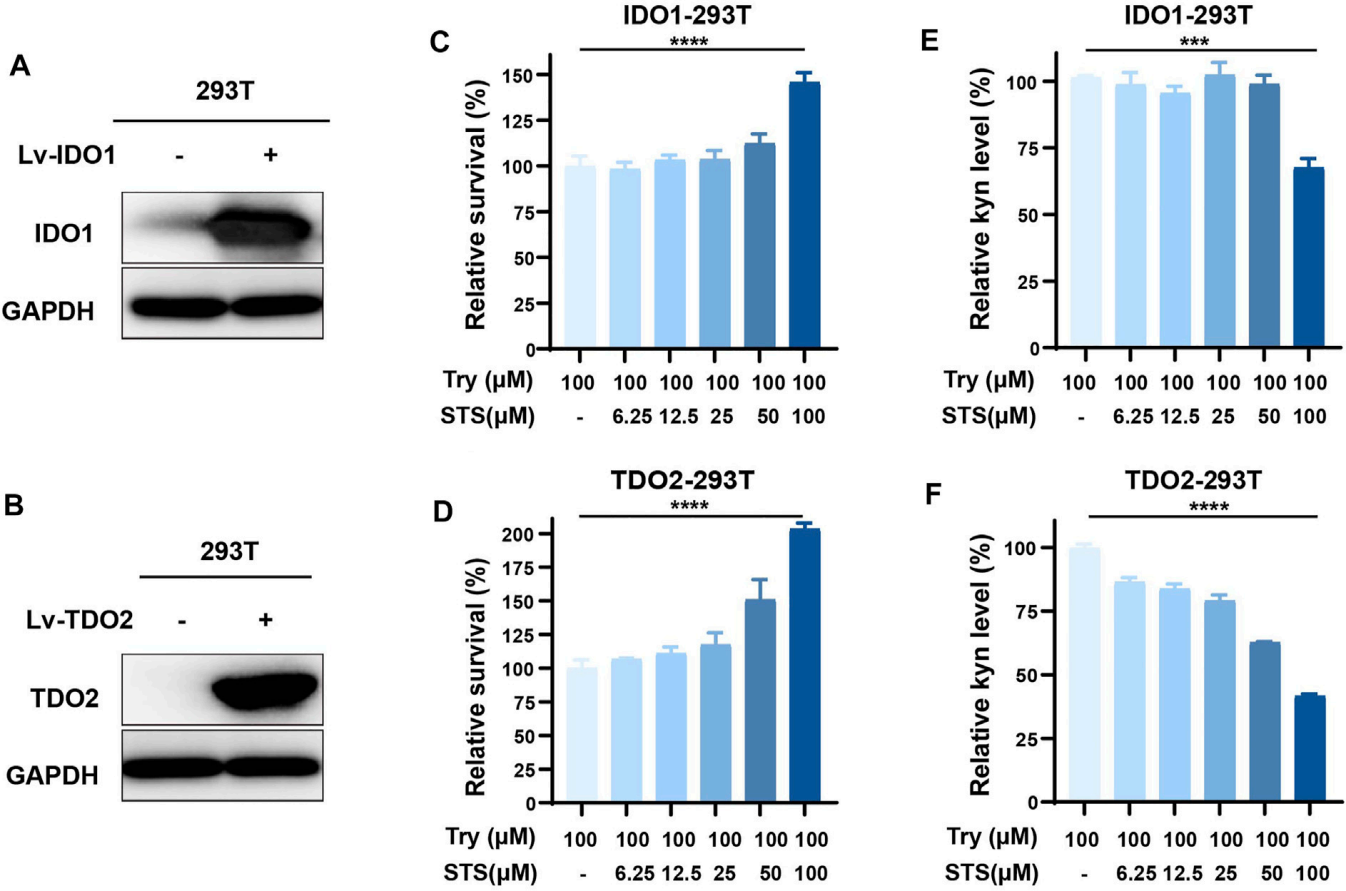
STS suppressed IDO1 and TDO2 activities intracellularly. **(A)** Overexpression of IDO1 in 293T cells. Western blot analysis was used to investigate protein expression level after transfecting the overexpression lentivirus for 48 h. Lv-IDO1 was the overexpressing lentivirus. **(B)** Overexpression of TDO2 in 293T cells. Western blot analysis was used to investigate protein expression level after transfecting the overexpression lentivirus for 48 h. Lv-TDO2 was the overexpression lentivirus. **(C,D)** The effect of STS on the proliferation of IDO1-293T and TDO2-293T cells. **(E)** STS decreased the production of kyn in IDO1-293T cells. The effect of STS on cell growth was considered during data analysis. **(F)** STS decreased the production of kyn in TDO2-293T cells. The effect of STS on cell growth was considered during data analysis. After the kyn was detected in the medium, the cell proliferation was measured by MTT assays. Statistical data are represented as the mean ± SD. ***p < 0.001 and ****p < 0.0001 analyzed by ANOVA.

To detect the effect of STS on IDO1 and TDO2 activities at the cellular level, we established the IDO1- or TDO2-overexpressing cell lines IDO1-293T and TDO2-293T by the lentiviral introduction of vectors to further validate the dual inhibitory activity of STS against IDO1 and TDO2. After 48 h lentiviral transfection, the expression levels of IDO1 and TDO2 were increased compared with the control. We also noted that the primary expression levels of IDO1 and TDO2 in the 293T cells were weak compared with those in overexpressing cells by WB (Figures 2A,B). The presence of kynurenine in the culture medium further confirmed this conclusion (Supplementary Figure S2). When kynurenine was detected in the medium, we discovered that STS had a significant promoting effect on the proliferation of IDO1- or TDO2-overexpressing 239T cells, particularly at high concentrations (Figures 2C,D). In the IDO1-293T and TDO2-293T cell culture medium, the relative level of kynurenine was impeded by 24 h of 100 μM STS treatment after considering the impact of STS on cell proliferation when we measured the level of kynurenine in the medium (Figures 2E,F).

### Sodium Tanshinone IIA Sulfonate Reduced the Proportion of Tregs in Lymphocytes and Scarcely Affected Lymphocyte Proliferation

**FIGURE 3:**
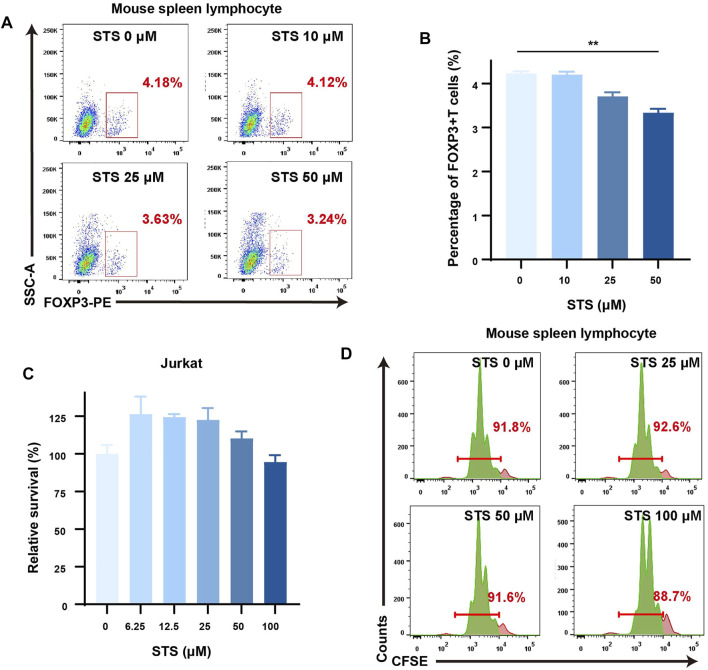
STS reduced the percentage of Treg cells with a slight effect on the proliferation of lymphocytes. **(A)** STS decreased the percentage of Tregs. Flow cytometry was employed to analyze the percentage of Tregs in the coculture system of murine spleen lymphocytes and CT26 after STS treatment for 48 h. The gate of Tregs was from Zombie^−^ CD45+ CD4+ cells. **(B)** Quantification of the percentage of Tregs. **(C,D)** STS had little effect on the proliferation of lymphocytes. Survival diagram of Jurkat cells after treatment with STS for 72 h **(C)**. CFSE staining was used to analyze the influence on the proliferation of mouse splenic lymphocytes after 48 h of treatment with STS by flow cytometry **(D)**. Statistical data are represented as the mean ± SD. **p < 0.01 analyzed by ANOVA.

It has been proven that kynurenine from tryptophan metabolism in cancer cells can induce the expression of FOXP3, a marker of regulatory T cells (Tregs), by activating the AhR nuclear transform to suppress tumor immunotherapy (Roncador et al., 2005). We wondered whether STS could weaken the effect of tumor cells on Treg production through the IDO-kynurenine-AhR axis *in vitro*. To be clear, we utilized a cocultured system of CT26 and mouse spleen lymphocytes to preliminary mimic the effect of tumor cells on T cells. Prior to this, we used the MTT assay to examine the growth of CT26 cells after 72 h of STS administration and observed that STS had little effect on CT26 cell growth (Supplementary Figure S3). Then, after 48 h of STS treatment, flow cytometric analysis showed that STS (50 μM) could lower the percentage of FOXP3+ CD4+ T cells in the cocultured system (Figures 3A,B).

TSN has been reported to have antioxidative, anti-inflammatory, and cytotoxic properties in multiple types of human cancer cells (Su et al., 2008). In addition, TSN can decrease lymphocyte proliferation (Zhang et al., 2016). As a result, it is worth considering whether STS can inhibit lymphocyte cell proliferation similar to TSN. To make it clear, we first assessed the effect of STS on Jurkat cell proliferation by MTT assay. According to our findings, even at high concentrations (100 μM), STS treatment for 72 h had only a minor effect on Jurkat cell proliferation (Figure 3C). Furthermore, the lymphocyte CFSE staining assay showed a similar result: after treatment for 48 h, STS scarcely affected the proliferation of the lymphocytes (Figure 3D).

### Sodium Tanshinone IIA Sulfonate Improved the Antitumor Activity of the Anti-PD1 Therapy *In Vivo*

**FIGURE 4:**
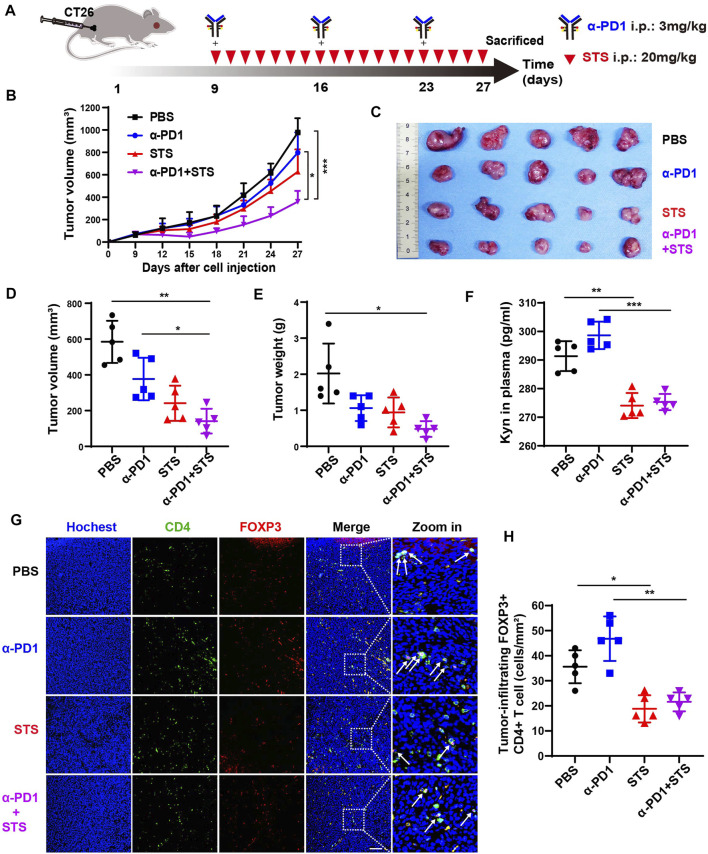
STS elevated the efficacy of anti-PD1 to inhibit CRC progression by decreasing the number of Tregs. **(A)** Schematic model illuminating methods of the murine CRC model establishment and therapy. Anti-PD1 (α-PD1, 3 mg/kg): i.p. once a week. STS (20 mg/kg): i.p. once a day. The mice were sacrificed on day 27. **(B)** The average tumor growth curves for mice treated with phosphate-buffered saline (PBS), α-PD1, STS, and α-PD1 plus STS. The tumor volume was measured every 2 days. **(C–E)** The photograph, volum and weight of tumors on day 27. **(F)** Concentrations of kyn in plasma. It was measured by ELISA. **(G)** Immunofluorescence analysis of tumor-infiltrating CD4+ T cells and Treg (FOXP3+ CD4+) cells after therapy. Scale bar: 100 μm. **(H)** Statistical analysis of the number of FOXP3+ CD4+ T cells in each group under a microscope at ×200 magnification. Statistical data are represented as the mean ± SD. *p < 0.05, **p < 0.01 and ***p < 0.001 analyzed by ANOVA.

Several publications have reported that TSN inhibits tumor growth *in vivo*, including in CRC (Wang et al., 2005; Su and Lin, 2008; Chiu et al., 2013). However, there is no evidence indicating that STS inhibits tumor development *in vivo*. To investigate the effect of STS on tumor growth *in vivo*, we developed a murine model of CRC *via* subcutaneous injection of CT26 cells. When the tumor volume reached ≈80 mm^3^, the mice were randomly assigned to four therapy groups (*n* = 5): PBS; STS; anti-PD1; and STS + anti-PD1. The dosage of anti-PD1 (3 mg/kg, once a week) was according to the library (Liu et al., 2019) with a minor modification. All the treatments were intraperitoneal injections (Figure 4A), and the tumor volumes were measured every 2 days. The mice were euthanized on day 27, and the excised tumors were photographed, measured in volume, and weighed. The tumor growth curves showed that STS monotherapy better suppressed tumor development than PBS and enhanced therapeutic effect of anti-PD1 (Figure 4B). As expected, the curative effect of combined therapy with STS plus anti-PD1 was significantly better than other therapies in retarding tumor development in terms of tumor volume and weight (Figures 4C–E).

### Sodium Tanshinone IIA Sulfonate Enhanced Tumor Immunotherapy by Decreasing Treg Numbers and Increasing CD8+ T Cell Numbers in Tumors

After anti-PD1 therapy, the plasma kynurenine level of melanoma and renal cancer patients are increased (Li et al., 2019a). This phenomenon was also observed in our results. In plasma, the level of kynurenine from anti-PD1 therapy was partially increased compared with PBS (Figure 4F). As predicted, compared with anti-PD1 monotherapy, comnination with STS had a lower amount of kynurenine in plasma (Figure 4F).

Kynurenine raises the quantity of Tregs, which harms the effective of immune response, as is generally accepted (Cheong and Sun, 2018). Therefore, decreasing Tregs may be commendably benefit to tumor immunotherapy (Munn et al., 2018; Tanaka and Sakaguchi, 2019). We accordingly investigated the number of Tregs in tumors after therapy. Compared with anti-PD1 monotherapy, STS decreased the number of FOXP3+ CD4+ T cells in combination therapy (Figures 4G,H).

**FIGURE 5:**
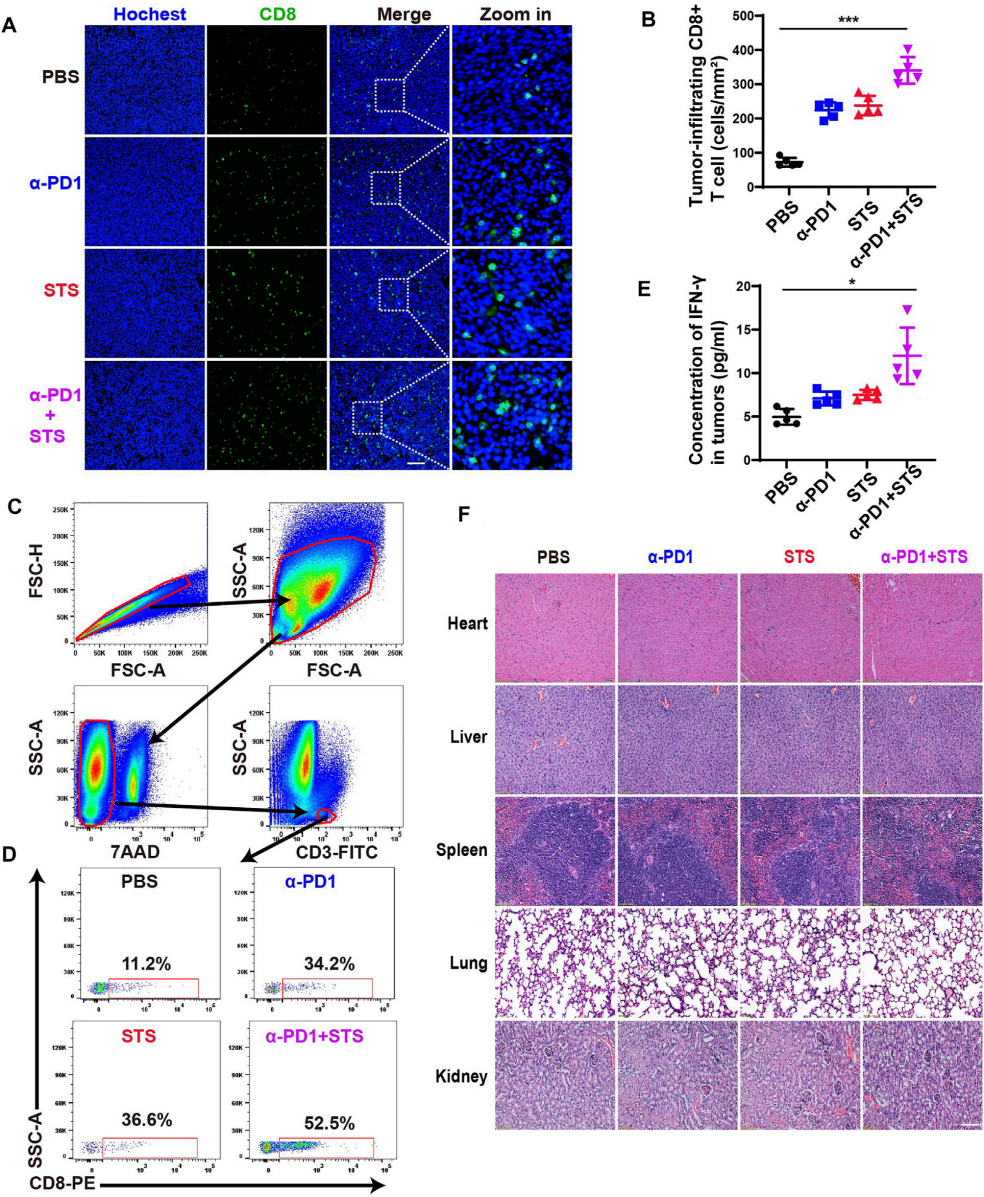
STS improved the therapeutic effect of anti-PD1 by increasing the number of CD8^+^ T cells and the concentration of IFN-*γ* in tumors. **(A)** Immunofluorescence analysis of tumor-infiltrating CD8+ T cells after therapy. Scale bar: 100 μm. **(B)** Statistical analysis of the number of CD8+ T cells in each group under a microscope at 200x magnification. **(C,D)** The percentage of CD8+ T lymphocytes in all CD3+ T cells in tumors after therapy. **(E)** The concentration of IFN-*γ* in tumor tissues. It was detected by ELISA. **(F)** Hematoxylin and eosin (H&E) staining of the main organs of mice. Scale bar: 100 μm. Statistical data are represented as the mean ± SD. *p < 0.05 and ***p < 0.001 analyzed by ANOVA.

The number of tumor-infiltrating CD8+ T cells (TILs) is closely related to the degree of response to immunotherapy (Dahlin et al., 2011; Paijens et al., 2021). We intended to explore whether STS therapy could increase the number of CD8+ T cells in tumors. Immunofluorescence assay of tumor tissues revealed that the combination therapy group had the maximum numbers of CD8+ T cells compared with the other three groups (Figures 5A,B). Flow cytometry also showed consistent results (Figures 5C,D). Furthermore, IFN-*γ* in tumor tissues also plays an important role in tumor immunotherapy. Therefore, we also detected the level of IFN-*γ* in tumor tissues by ELISA. A higher IFN-*γ* concentration was observed in STS and anti-PD1 combination therapy (Figure 5E). The findings mentioned demonstrated that STS could enhance immunotherapy for CRC.

In addition, we stained and inspected the main organs (heart, liver, spleen, lung, and kidney) of mice using hematoxylin and eosin (H&E) to evaluate the toxicity *in vivo*. There was no indication that STS, either alone or in combination with anti-PD1, produced obvious organ damage (Figure 5F).

## Discussion

Tanshinone IIA sulfonic acid sodium salt is a material for a drug named sulfotanshinone sodium injection, which has been used to treat heart failure over the past years in China. It was developed to increase the hydrophilicity of TSN and retains some of TSN’s pharmacological properties, such as anti-inflammatory and cardioprotective properties. However, TSN has been reported to suppress the proliferation of tumor cells by inducing apoptosis and autophagy, while STS has not yet been proven to suppress tumor cell growth. All the above results suggest several differences between TSN and STS. There were also some distinctions between the two compounds in the inhibitory activities of IDO1 and TDO2 in our results. As proven in previous research, STS had obvious inhibitory activity on IDO1, but TSN did not (Zhao et al., 2019). Furthermore, STS possessed higher inhibitory activity on TDO2 than TSN as the presence of sulfonic acid group. In molecular docking, the amino acid residues interacting with the sulfonic acid group of STS were important for IDO1 and TDO2 catalytic activity. It seems that the sulfonic acid group of STS plays a crucial role in improving the inhibitory activity on IDO1 and TDO2.

IDO1 is highly expressed in multiple tumor types, including melanoma, lung cancer, pancreatic cancer, and renal cell carcinoma (Li et al., 2019b). Previous studies have found that IDO1 promotes tumor immune escape (Friberg et al., 2002). Its activity is employed as an important predictor of immunotherapy response (Ferns et al., 2015; Liu et al., 2016; Seeber et al., 2018), and the inhibitors of IDO1 are widely studied in tumor combination therapy (Peng et al., 2018; Zhang et al., 2020). In CRC, plasma IDO1 activity can be used as a prognostic biomarker (Cavia-Saiz et al., 2014). IDO1 inhibitors combined with radiotherapy, chemotherapy, or immunotherapy can delay tumor progression by reducing the generation of kynurenine and increasing the cytotoxicity of T cells in murine models of CRC (Jia et al., 2018; Jung et al., 2019; Liu et al., 2019). In our findings, as a dual inhibitor of IDO1 and TDO2, STS could lower the level of kynurenine in the plasma and leaded an increase in the number of CD8+ T cells in tumors. Treg, which can be induced by kynurenine, is a key immunosuppressive cell in tumors, primarily secreting cytokines such as IL-10, IL-35, and TGF-*β* (Takahashi et al., 1998; Collison et al., 2007). Furthermore, cytotoxic molecules generated by Treg cells, such as perforin and granzyme, can destroy effector T cells (Cao et al., 2007). As a result, targeted inhibition of Treg cells can effectively boost tumor immunotherapy (Ohue and Nishikawa, 2019). In our experiment, STS diminished the Treg numbers in the tumor microenvironment.

Existing tumor immunotherapy strategies primarily activate tumor-specific immune responses, such as increasing the cytotoxicity of CD8+ T cells or natural killer cells (NK cells) (Farhood et al., 2019). For example, anti-PD1 therapy can alleviate tumor-induced immunosuppression of T lymphocytes in melanoma and kidney cancer. However, after anti-PD1 therapy, activated T cells produce large amounts of IFN-*γ* which inversely inhibits T cell activity by IDO-induced tryptophan depletion. This may be one of the causes of acquired resistance to anti-PD1 therapy. In our short investigation, STS extended the response to anti-PD1 therapy. Further studies on whether STS could maintain the long-term response to immunotherapy are awaited.

Although anti-PD1 therapy is widely applied in immunotherapy with encouraging results, the adverse effects, such as myocarditis and colitis (Sullivan and Weber, 2021), should not be ignored. The cardioprotective and anti-inflammatory properties of STS may help patients benefit more from immunotherapy. On the other hand, STS has also been proven to be proangiogenic. It is well accepted that neovascularization plays a critical role in tumor development, invasion, metastasis, and resistance to therapy (Xu et al., 2022). Moreover, angiogenesis inhibitors combined with ICB show remarkable antitumor efficacy in non-small cell lung cancer, hepatocellular carcinoma, and CRC (Schmittnaegel et al., 2017; Zhu et al., 2021). Therefore, our further study will focus on whether STS with angiogenesis inhibitors and ICB can be a more effective therapeutic strategy for CRC.

## Data Availability

The original contributions presented in the study are included in the article/Supplementary Material, further inquiries can be directed to the corresponding authors.
