# Supplementary material for: Sodium Tanshinone IIA Sulfonate as a Potent IDO1/TDO2 Dual Inhibitor Enhances Anti-PD1 Therapy for Colorectal Cancer in Mice
Source: Front Pharmacol. 2022 Apr 27;13:870848. doi: 10.3389/fphar.2022.870848 (PMC9091350; doi:10.3389/fphar.2022.870848)
Supplement: Supplementary file 1 [file DataSheet1.pdf]

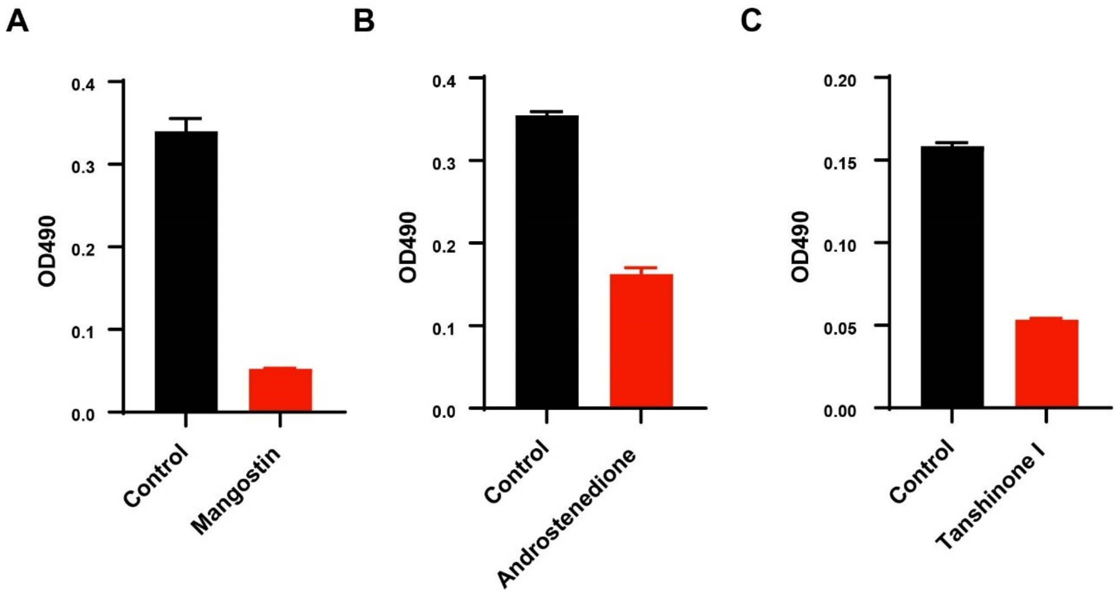

FIGURE S1 The activity compounds in drug screening for IDO1 and TDO2 inhibitors. (A,B) The IDO1 activity after mangosetin or androstenedione addition was detected at 490 nm. (C) The TDO2 activity after Tanshinone I addition was detected at 490 nm. The concentration of compounds was 10 μM. For each compound, there are two replicated wells in drugs screening. The data are presented as mean ± SD.

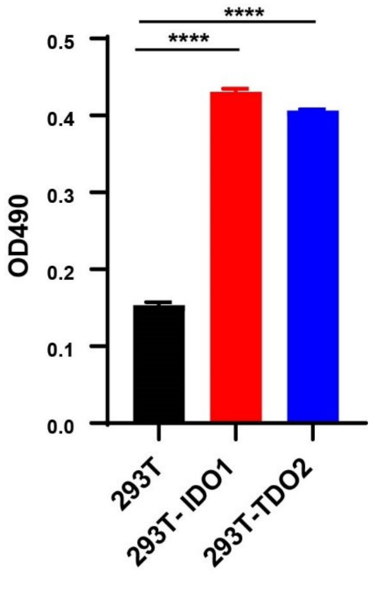

FIGURE S2 IDO1/TDO2 overexpression increases the cellular kynurenine level. After overexpressing IDO1 or TDO2 in 293T cells, the kynurenine level was increased by detecting the value of OD490. Four replicated wells were set. The data are presented as mean ± SD. ****P < 0.0001 analyzed by ANOVA.

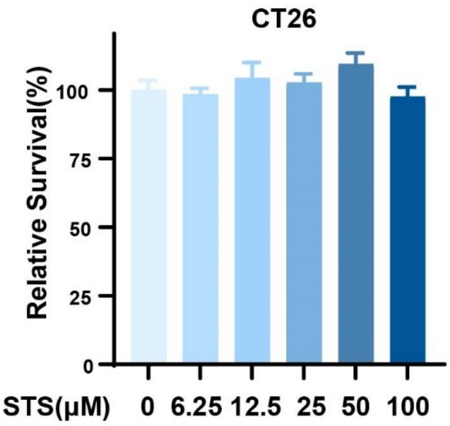

FIGURE S3 STS does not affect the proliferation of CT26 cells. Each concentration of STS had five replicated wells. The data are presented as mean ± SD.
